# Longitudinal Assessment of ROX and HACOR Scores to Predict Non-Invasive Ventilation Failure in Patients with SARS-CoV-2 Pneumonia

**DOI:** 10.2478/jccm-2024-0013

**Published:** 2024-04-30

**Authors:** Abhijeet Anand, Sai Teja Kodamanchili, Ankur Joshi, Rajnish Joshi, Jai Prakash Sharma, Goyal Abhishek, Abhijit P Pakhare, Yogesh Niwariya, Rajesh Panda, Sunaina T Karna, Alkesh K Khurana, Saurabh Saigal

**Affiliations:** All India Institute of Medical Science, Bhopal, Madhya Pradesh, India; Community and Family Medicine, All India Institute of Medical Science, Bhopal, Madhya Pradesh, India; Department of Anaesthesia and Critical Care, All India Institute of Medical Science, Bhopal, Madhya Pradesh, India; Department of Pulmonary Medicine, All India Institute of Medical Science, Bhopal, Madhya Pradesh, India; Department of Cardiothoracic Vascular Surgery, All India Institute of Medical Science, Bhopal, Madhya Pradesh, India; Kalinga Institute of Medical Sciences, Bhubneshwar, India

**Keywords:** non-invasive ventilation, HACOR, ROX, COVID-19, ARDS

## Abstract

**Introduction:**

NIV (Non-invasive ventilation) and HFNC (High Flow nasal cannula) are being used in patients with acute respiratory failure. HACOR score has been exclusively calculated for patients on NIV, on other hand ROX index is being used for patients on HFNC. This is first study where ROX index has been used in patients on NIV to predict failure.

**Aim of the study:**

This study investigates the comparative diagnostic performance of HACOR score and ROX index to predict NIV failure.

**Methods:**

We performed a retrospective cohort study of non-invasively ventilated COVID-19 patients admitted between 1st April 2020 to 15th June 2021 to ICU of a tertiary care teaching hospital located in Central India. We assessed factors responsible for NIV failure, and whether these scores HACOR/ROX index have discriminative capacity to predict risk of invasive mechanical ventilation.

**Results:**

Of the 441 patients included in the current study, 179 (40.5%) recovered, while remaining 262 (59.4%) had NIV failure. On multivariable analysis, ROX index > 4.47 was found protective for NIV-failure (OR 0.15 (95% CI 0.03–0.23; p<0.001). Age > 60 years and SOFA score were other significant independent predictors of NIV-failure. The AUC for prediction of failure rises from 0.84 to 0.94 from day 1 to day 3 for ROX index and from 0.79 to 0.92 for HACOR score in the same period, hence ROX score was non-inferior to HACOR score in current study. DeLong's test for two correlated ROC curves had insignificant difference expect day-1 (D1: 0.03 to 0.08; p=3.191e-05, D2: −0.002 to 0.02; p = 0.2671, D3: −0.003 to 0.04; p= 0.1065).

**Conclusion:**

ROX score of 4.47 at day-3 consists of good discriminatory capacity to predict NIV failure. Considering its non-inferiority to HACOR score, the ROX score can be used in patients with acute respiratory failure who are on NIV.

## Introduction

SARS-CoV-2 infection has wide range of clinical manifestations. Severe pneumonia leading to acute respiratory distress syndrome (ARDS) is one of the most catastrophic manifestation having high mortality [1]. ARDS presents as quickly escalating hypoxemia with a significant ventilation-perfusion mismatch [2]. Lung compliance is preserved early in ARDS, and as the disease progresses, lung compliance is reduced due to worsening alveolar-oedema and fibrosis [2].. Prior to SARS-CoV-2 pandemic, traditional approach for management of ARDS has been to intubate and perform invasive, lung-protective ventilation with proning. However during SARS-CoV-2 pandemic large numbers, limitation in resources and avoidance of endotracheal intubation prompted a large number of patients to be managed by non-invasive ventilation [3].

Non-invasive ventilation (NIV) is administered either by a high-pressure or a high-flow system. High-pressure systems depend on a specialized NIV-mask and deliver positive end-expiratory pressure (PEEP), which increases oxygenation [4,5]. In-contrast, high-flow systems depend on a specialized nasal canula (HFNC) and delivers oxygen and air at a high flow rate. Both methods reduce work of breathing and assist inspiration. Since assisted ventilation is often required for a prolonged duration and use of either of these techniques depends on availability and patient-comfort, often both these techniques are used alternately in a same patient. Failure of NIV to correct hypoxemia may lead to intubation and invasive mechanical ventilation (IMV) [6,7,8,9].

The incremental duration of non-invasive ventilation (NIV failure) has a direct association with self-inflicted lung injury which further leads to worsening of respiratory mechanics and poor outcome [10–11. HACOR score (heart-rate, acidosis, consciousness, oxygenation, and respiratory rate) was initially proposed for NIV failure [10]. A more simplified ROX index (ratio of respiratory rate and oxygenation) was developed for prediction of high-flow failure [12].

Prior to COVID-19 era, HACOR score was exclusively calculated for patients on NIV failure and ROX index was exclusively calculated for HNFC patients.. During the COVID-19 pneumonia Valencia et al exclusively compared these score in patients requiring HFNC [13]. We in this study explore further comparative performance of these two indices in NIV failure from longitudinal perspective. The secondary objectives of the study were to detect the optimum cut-off values of superior indicator and to check the effect of other co-variables on NIV status from the multivariable perspective after adjusting the effect of superior indicator(ROX/HACOR).

## Material and Methods

### Design and Ethics statement

We performed a retrospective cohort study of noninvasively ventilated COVID-19 patients admitted to an intensive care unit (ICU) of a tertiary care teaching hospital located in Central India. During the study period COVID-ICU was operated under trained intensivists, who recorded all patient details on ICU-charts. A hospital information system recorded patient demographics and laboratory based investigation details. As part of standard hospital practice, all patients presenting to the emergency area of the hospital were triaged and those with severe illness (SpO_2_< 90% on room air) were considered for ICU admission. Patients admitted to ICU were managed with one of the modalities namely NIV, HFN C, O2 therapy. Patients who had either tachypnea (RR > 30 per minute) or a high oxygen demand (FiO2 more than 0.60) were considered for invasive mechanical ventilation.. A request for waiver of consent was approved by Institutional Ethical Committee of AIIMS Bhopal (2020/DM/Mch/July/01).

### Participants

We included case records of all adult COVID-19 RTPCR positive patients who were admitted between 1^st^ April 2020 to 15^th^ June 2021 in COVID-ICU on noninvasive ventilation as an initial ventilatory strategy. We excluded patients where a an early decision of IMV (within 24 hours of ICU admission), pregnant women, and patients transferred to other facilities on request.

#### Study Procedures

We abstracted information pertaining to demography (age, gender), pre-admission morbidity (Diabetes mellitus, hypertension, ischemic heart disease, chronic kidney disease, malignancies etc.) COVID-19 related disease history (onset and nature of symptoms, date of admission to hospital and admission to ICU), vitals, oxygenation and SOFA score at the time of ICU admission. Based on available information HACOR score/ROX index were calculated 8 hourly for the duration of 6 days or until NIV failure. Out of 3 scores calculated per day we used the worst HACOR score/ROX index for the purpose of the study. The date of intubation and onset of mechanical ventilation, parameters related to mechanical ventilation related mechanics, investigations, administration of key therapeutic agents, and outcome during hospital stay were also retrieved from ICU charts.

### Outcome

Key outcome was NIV-failure, which was defined as initiation of IMV or death while patient was on NIV. Other operational definitions used in the study are further described in *Supplementary appendix-S1.*

### Statistical analysis

We entered all data in MS-Excel, and performed data-cleaning before exporting to statistical analysis software R(RStudio 2022.02.0+443, 2022-02-16). We performed a descriptive statistical analysis of all variables, with NIV-failure as a key-outcome. We estimated 95% confidence intervals for all point estimates. We used box plots, violin plots and ribbon plots to check the trajectory of ROX and HACOR scores. To evaluate performance of HACOR score and ROX index, we considered ICU admission as day-0 and constructed ROC-curves for both these parameters for every consecutive day. Patients who had an outcome prior to day-6 were right censored.

Two composite Receiver Operating Characteristic (ROCs) curves for ROX and HACOR scores were created for each day and AUC with 95% confidence interval for each day was calculated. The cut off points (with confidence regions) on these graphs were calculated using the Clopper and Pearson exact method and the cross product of these intervals drew the rectangular confidence interval for the pair. The ROC plotting was also done in pairwise manner (ROX and HACOR score for the day) for visual comparison of scores to predict day wise NIV status. The extent of superposition was detected through Venkatraman method with default boot-strapping value as 2000.

The day wise optimum cut-off point of score to discriminate NIV failure to NIV success were calculated through 4 different methods in R-package “Optimal Cutpoints. The rationale to choose these methods, amongst given methods of calculating cut points was based on - a) to assign relatively higher sensitivity and NPV to cut offs at both population levels and an clinical decision making at an individual level b) a mix of deductive and inductive (Bayesian) approaches and c) incorporation of both novel and time honoured method. Thus we chose Positive Diagnostic Likelihood Method (desired DLR+ set to 10), minimum sensitivity method (where desired sensitivity was pre-set to 90%), Negative Predictive Value maximization method and Yoden Index. The day wise cut off with corresponding sensitivity, specificity, NPV and PPV for that cut off value was calculated. Choosing and optimizing cut off through multiple methods assigned an intrinsic validity to cut-off value. A composite visualization of bar and line geometry was then created in order to see the day wise cut off values (represented by bars at secondary y-axis) and corresponding parameters values (represented by different lines at primary y-axis). The purpose was to gather other visual evidence on the day at which ROX cut off values will maximize the prediction as a function of time.

We had chosen 4 models to understand the effect of overall marginal ROX score, interaction of ROX score with day and effects of other covariables in multivariate sense. These models were built with gradual increasing intricacies arising from theory-driven variable selections The schematic description is given in *Supplementary appendix-S2*. In these models, NIV-failure was used as a key outcome variables and demographic, clinical, SOFA score, haematological, and biochemical parameters at baseline as predictors. Performance of multivariable models was assessed using Akaike information and baysean information criteria. The visualization of marginal effects for complex models were made by ‘ggeffect’ package in r which computes marginal effects and accommodative predictors (or *estimated marginal means*) at the mean (MEM) or at representative values (MER) of predictors from statistical models by keeping the non-focal variables constant and changing the focal variables. The resultant data frame with consistent structure was then used for plotting using “ggplot”. The singular and adjusted effects of the significant variables were plotted through probability distribution plots.

## Results

**Fig. 1.**
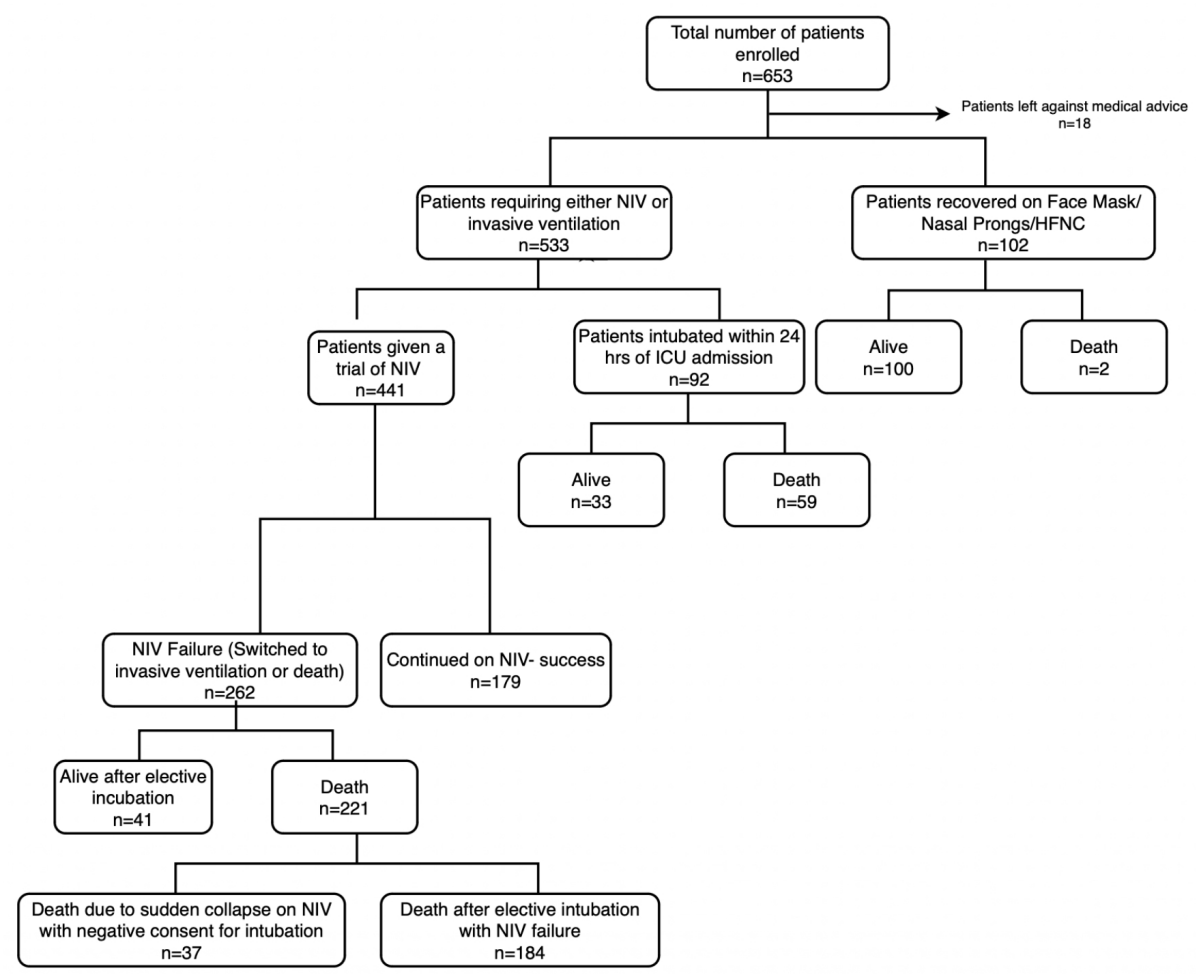
Study flow chart

Out of 653 ICU admissions between 1^st^ April 2020 to 15^th^ June 2021, 441 (67.5%) received NIV as initial ventilatory strategy for >24 hours. While 92 (14.0%) were intubated and mechanically ventilated within 24 hours of ICU admission and another 102 (15.6%) recovered on oxygenation alone. Some patients left against medical advice. A total of 262 patients had NIV failure and were shifted either to invasive ventilation or died during NIV. The whole study flow is summarized in Figure 1 IMV. Individuals with NIV-failure had a higher age, more severe ARDS, higher SOFA scores, and a greater prevalence of Acute Kidney Injury, hypotension and vasopressor use as compared to those who did not fail NIV. Individuals with no NIV-failure received more steroids and had a lower length of ICU stay (Table 1).

**Table 1.** Comparison of Demographic, Severity of illness, Inflammatory markers between NIV success vs Failure patients

	**Total**	**NIV Success (n=179)**	**NIV failure (n=262)**	**p value**
Age	60 (19–90)	54(53–58)	62(61–64)	<0.001
M:F	311:130	126:53	185:77	0.960
DM	241(54.6%)	106(59.2%)	135(56%)	0.111
HTN	268(60.8%)	103(57.5%)	165(63%)	0.251
CAD	64 (14.5%)	22(12.3%)	42(16%)	0.274
CKD	24(5.4%)	7(3.9%)	17(6.5%)	0.241
COAD	22(5%)	8(4.5%)	14(5.3%)	0.629
Symptom-admission duration (days)	7 (0–14)	3(0–8)	4(1–14)	0.783
Baseline P/F ratio	125(56–280)	145(140–156)	117(110–121)	<0.001

**ARDS category**				<0.001
Mild	32 (7.3%)	18(56.3%)	14(43.8%)	
Moderate	282 (63.4%)	136(48.2%)	146(51.8%)	
Severe	127(28.8%)	25(19.7%)	80.3%	
Remedesivir	286 (64.9%)	117(65.4%)	169(64.5%)	0.853
TCZ	23 (5.2%)	11(6.1%)	12(4.6%)	0.468
Vasopressors	172(39%)	2(1.1%)	170(64.9%)	<0.001

**Steroid**				<0.001
Early High	283 (64.2%)	126(70.4%)	157(59.9%)	
Early low	113 (25.6%)	50(27.91%)	63(14.3%)	
Indeterminate	45 (10.2%)	3 (1.7%)	42(9.5%)	

**BSI**				
NG	351(79.6%)	164(91.6%)	187(71.4%)	<0.001
Gram neg	56(12.7%)	9(5%)	47(17.9%)	
Positive	14 (3.2%)	3(1.7%)	11(4.2%)	
Fungal	20(4.5%)	3(1.7%)	17(6.5%)	
Shock	134 (30.4%)	1(0.6%)	133(50.8%)	<0.001
AKI	109(24.7%0	8(4.5%)	101(38.5%)	<0.001
SOFA	4(2–14)	3(3–4)	6(6–7)	0.0001
Baseline CRP	101(1.98–870)	75(68–98.70)	116.20(98–135)	0.006
Baseline ALC	760(120–3640)	860(760–920)	720(650–780)	<0.001
ICU LOS	9(1–90)	7(7–8)	11(10–13)	<0.001
Hospital LOS	14(1–95)	14(12–16)	14(13–15)	0.349

**Fig. 2.**
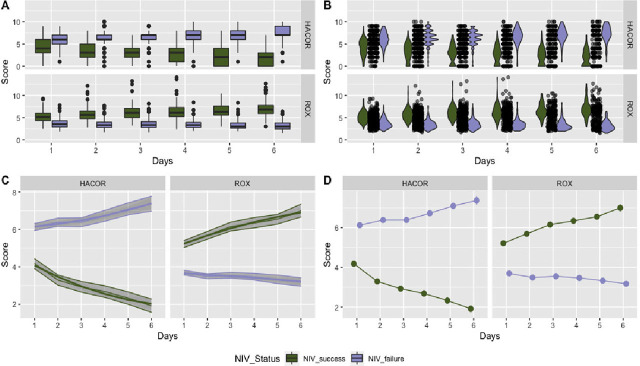
Patterns of ROX index and HACOR from day 1 to day 6

**Fig. 3.**
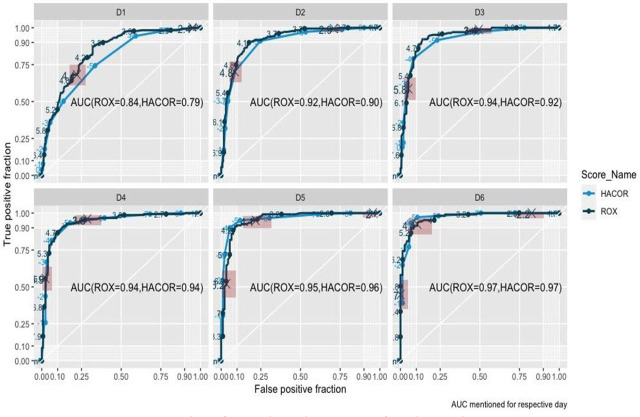
Receiver operating curve analysis of ROX-index and HACOR score from day 1 to day 6

Performance of both HACOR score and ROX index was found to be similar. Individuals who had NIV-failure had a progressive rise in HACOR score, and a corresponding decline in ROX-index from day 1 to day 6 (Table 2). The similar or non-inferior discrimination of both HACOR score and ROX index between the NIV-failures and NIV-success subgroups in visually depicted in Figure 2. ROC analysis further corroborates to this finding. The area-under-curve for prediction of failure rises from 0.84 to 0.94 from day 1 to day 3 for ROX index and from 0.79 to 0.92 for HACOR score in the same period while further increment in predictiveness is marginal for both scores from day 4 to 6 (Figure 3). DeLong's test for two correlated ROC curves also had an insignificant difference expect on day-1 (D1: 0.03 to 0.08; p=3.191e-05, D2: −0.002 to 0.02; p = .2671, D3: −0.003 to 0.04; p= 0.1065, D4: −0.01 to 0.02; p= 0.7326, D5: −0.03 to 0.02; p= 0.5062, D6: −0.01 to 0.02; p= 0.9423).

**Table 2.** Ventilatory parameter comparison- HACOR/ROX

	**Total**	**Success (n=175)**	**Failure (n=262)**	**Univariate analysis OR (CI)**
HACOR D1	5(0–9)	4(4–5)	6(6–7)	1.963(1.696–2.273)
ROX D1	4.13(1.9–9.27)	5.14(4.9–5.3)	3.53(3.33–3.68)	0.309(0.245–0.389)
HACOR D2	5(0–10)	3(3–4)	6(6–7)	2.735(2.250–3.325)
ROX D2	4.1(1.7–12.15)	5.62(5.36–5.85)	3.32(3.16–3.50)	0.194(0.143–0.262)
HACOR D3	5(0–9)	3(3–4)	7(7–8)	3.064(2.435–3.857)
ROX D3	4.5(1.68–13.22)	6.06(5.85–6.27)	3.3(3.13–3.50)	0.186(0.131–0.263)
HACOR D4	5(0–10)	3(3–4)	7(7–8)	3.119(2.416–4.027)
ROX D4	2.7(2–14.1)	6.08(5.83–6.50)	3.29(3.10–3.57)	0.182(0.125–0.266)
HACOR D5	4.0(0–10)	2(2–3)	7(7–8)	3.745(2.647–5.300)
ROX D5	5.2(1.95–10.47)	6.3(6.15–6.6)	2.95(2.8–3.20)	0.182(0.119–0.279)
HACOR D6	4 (0–10)	2.0(2–3)	7.5(7–8)	3.536(2.442–5.119)
ROX D6	5.4(1.5–12.65)	6.84(6.35–7.14)	2.89(2.66–3.20)	0.159(0.093–0.272)
No of days with HACOR>5	2(0–18)	1(1–2)	3(3–4)	1.87(1.58–2.09)

The optimum cut-off values for the ROX score to predict NIV failure was determined as 4.47 at day-3. The detailed description of the day wise optimum cut off points, corresponding to 4 selected methods and sensitivity, specificity, PPV, NPV and likelihood point estimates with 95% CI are given in *Supplementary appendix-S2.*

On multivariable analysis, ROX index with a cutoff of greater than 4.47 was found protective for NIV-failure (OR 0.15 (95% CI 0.03–0.23). Age more than 60 years and higher SOFA score were other significant independent predictors of NIV-failure (Table 3).

**Table 3.** Multivariable models for NIV failure. The models were created on 327 participants having complete information about all covariates. The model diagnostics indicate the substantial reduction in error variances after theory driven sequential sections of variables

**Predictors**	**Ref. Category. NIV_Success Odds Ratios CI p**														

	**OR**	**CI**	**p**	**OR**	**CI**	**p**	**OR**	**Cl**	**p**	**OR**	**CI**	**p**	**OR**	**CI**	**p**
R0X>4.47	0.19	0.13–26	<0.001	0,18	0.12–0.25	<0.001	0,18	0.12–0.25	<0.001	0,17	0.11–0.25	<0.001	0,15	0.09–0.23	<0.001
Age>60Yrs				5,	2.47–11.83	<0.001	5,	238–11.74	<0.001	5,	230–1158	<0.001	4,	1.66–10.18	0,003
GenderF				0,64	0.29–1.41	0,271	59	0.26–1.33	0,208	0,65	0.28–1.47	0,300	37	0.13–1.00	57
Diabetes+				0,61	0.29-1.27	0,184	59	0.28–1.25	0,170	59	0.27–1.26	0,176	55	023–1.30	0,175
Illness_ICU_IntervalT2(Int)							0,72	0.29–1.72	0,456	0,76	031–1.86	355	0,77	0.27–2.15	0,617
Illness_ICU_IntervalT3(High)							2,	0.87–5.25	0,100	222	0.90–5.62	0,086	3,	1.16–9.26	0,027
ALCDI_Tertile2(Int)										0,45	0.17–1.11	87	0,87	0.29–258	0,796
ALCDI_Tertile3(High)										0,43	0.16–1.07	0,071	0,75	0.26–2.16	585
SOFA Score													2,	1.60–2.71	<0.001

Observations	327			327			327			327			327		
R2 Tjur	393			0,636			0,646			0,654			0,729		
AIC	224,			209,			207,			206582			169,		
log-Likelihood	−110213			−99334			−96387			−94291			−74,		

OR: Odds ratio; CI: Confidence interval

**Fig. 4.**
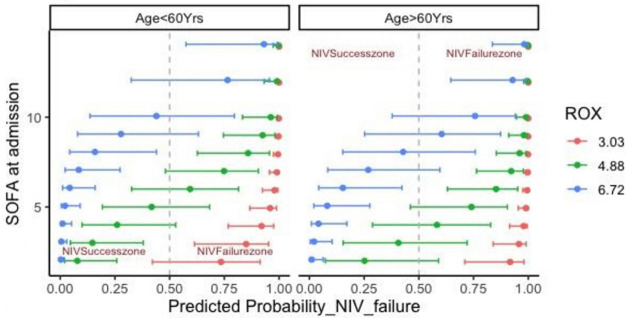
Age, SOFA, ROX score interaction

The visualization of marginal effects of age, SOFA and ROX score using probability distribution plots is shown in Figure 4. Older age independently seems to increase the probability of NIV failure irrespective of SOFA and ROX value. At older ages a relatively moderate SOFA score (8 or more) favours NIV failure even if ROX is on lower side. On the other hand, at younger ages a lower SOFA score with a moderate ROX favours NIV success. This observation may warrant the importance of estimating SOFA at admission amongst the relatively younger COVID-19 patients.

## Discussion

The HACOR score was developed by Duan et al where they observed an upward trends of HACOR score in patients with NIV failure [10]. The diagnostic accuracy for HACOR score of greater than 5, after 1 hour of NIV therapy was 81.8% and remained above 80% thereafter [10]. This accuracy was not found to be influenced with NIV duration, diagnosis, age, or disease. HACOR score is a summation based score where each indicative parameter is assigned a numerical value. A higher additive score thus indicates higher probability of NIV failure. On other hand, ROX index is a fraction based score where of SP02 to FiO2 ratio with respiratory rate as denominator [12]. With this context this study to the best of our knowledge is one of the largest single center series from India where 441 COVID-19 patients were initiated on NIV.

The faltering of the ROX is also evident in our study. In NIV failure group, amongst the subgroup having the ROX score less than 4.47 (and subsequently lending into failure) the ROX goes from 3.27± 0.59 (d1) to 3.23±0.66 (d3) to 2.94±0.72(d6). The subgroup having the ROX score greater than 4.47 in NIV failure group, the score trajectory was 5.26±0.70 (d1) to 5.54±1.01 (d3) to 5.48±0.65 (d6). In NIV success group, the subgroups having ROX scores above and below the identified cut-off values showed a score trajectory respectively from 5.83±1.00 (d1) to 6.35±1.39 (d3) to 7.28±1.67 (d6) and from 3.91±0.45 (d1) to 3.86 ±0.43 (d3) to 4.01±4.07 (d6). These descriptive statistics assigns a notion of stalling (faltering) courses of ROX in destined to be a NIV failure group. This stalling phenomenon holds true even for the subgroup erroneously misclassified as ‘normal’ based on identified ROX cut off in NIV failure group as well.

The discriminative capacity of HACOR was 0.79 (AUC-ROC-0.79) in our study; apart from study by Valencia et al (AUC-ROC-0.71); Santus et al (AUCROC-0.74); Guia et al (AUC ROC-0.87) the score has not been validated as per se in COVID-19 disease [13,14,15].

The ROX index greater than or equal to 4.88 measured after 12 hours of HFNC was significantly associated with a lower risk for MV (hazard ratio, 0.273 [95% confidence interval, 0.121–0.618]; p=0.002) [12]. Here in our study we calculated the cut off by three methods i.e. Direct logistic regression, 90% minimum sensitivity, high negative predictive value and by ROC, by all these methods ROX Cut off of 4.47 was >90% sensitive of predicting NIV failure. In recently published meta-analysis by JP et al., which included 1300 COVID-19 patients on HFNC therapy optimal cut off value for ROX index may fall close to 5 within 24 hours of admission with time to assessment was taken as 6 hours [16]. We in our study had a lower cutoff, this could be due to inclusion of patients with only on NIV therapy. These patients are much more hypoxemic than patients on HFNC and above all this cut off is for patients on NIV and not for patients on HFNC therapy. Other reason could be till now ROX scores have been computed in patients for a period of 48 hours but in our study we serially followed up to Day 6 where in patients with NIV failure, the serial ROX (48 vs 72 hours) would have a declining trend. AUC for ROX on D3 was equivalent to D6, hence Day 3 ROX would be better predictor of NIV failure. Logically also D3 seems fine once the patient is admitted to ICU, apart from respiratory support in our case it was NIV, anti-inflammatory therapy is initiated and by D3 it gives a clear picture whether the patient is improving or not and if not it's better to intubate as prolonging him on NIV for long time precipitates patient induced self-inflicted lung injury (SILI) [17].

In study by Valencia et al AUROC for ROX index was 0.72, HACOR was 0.71, these were marginally better than AUROC for respiratory rate 0.69 [13]. In meta-analysis by JP et al ROX index showed a good discriminatory value, sAUC 0.81 with sensitivity of 70% and specificity of 79% for predicting HFNC failure [16]. The reasons for non-inferiority of ROX index in patients with COVID-19 ARDS could be, the factors listed in HACOR score such as *HR, GCS and pH* if these factors are affected i.e. if sensorium is altered, tachycardia is due to shock/severe hypoxemia, or fall in pH is either due to respiratory acidosis or metabolic acidosis would eventually indicate that patient's needs mechanical ventilation within 24 hours. The criteria of pH was included in this score as this score was initially conceptualized for patients with acute exacerbation of COPD whereas in patients with COVID-19 pneumonia it's basically respiratory alkalosis which is troublesome rather than acidosis. We had eventually 92 patients who were immediately intubated i.e.<24 hours as they fitted into one of these criteria, hence were not included in final analysis. In COVID-19 patients the oxygenation and respiratory rates are usually affected whilst other parameters enlisted in HACOR score such as GCS, HR and pH are usually spared and if either of these three are involved then patient usually lands up in mechanical ventilation. This could be the reason leading to non-inferiority of ROX index in our study, and hence these scores should be interpreted with caution in other causes of respiratory failure besides COVID-19. The advantage of ROX index is it doesn’t require ABG and can be easily calculated bed side.

SOFA score was also one of the most important parameter on multimodal logistic regression. 75% of patients in current study had moderate to severe ARDS with a Respiratory SOFA of 3–4. A further increased SOFA would indicate renal or cardiovascular involvement, as CNS, coagulation SOFA and liver enzymes are relatively less affected. An indirect evidence of CVS and renal involvement may be thought off by higher vasopressor use and higher incidence of AKI in failure group. Age has been the significant factor which was associated with NIV failure, this has been demonstrated in study by Chacko et al [18]. Age has been associated with poor outcomes, various reasons could be poor host defense mechanisms, multiple comorbidities and poor cardiovascular response to hypoxemia, increased risk of AKI. A summary of the studies predicting NIV failure is illustrated in Table 4 [13,17,16,18,19,20,21].

**Table 4:** Summary of studies depicting NIV failure

**Reference, Year (Country)**	**Setting**	**Modes**	**NIRS failure definition**	**HACOR score in Failure**	**ROX Score in Failure**	**NIRS Failure**	**Mortality**	**Predictors of Failure**
Duan et al. 2021 (China)^19^	ICU (non-COPD)	NIV	Need of IMV as rescue therapy	7 (at initiation) 5 (at 1–2 hours)		14.7 %	9.4 %	HACOR score, Duration of NIV, Heart rate, pH, GCS, Mean Arterial BP, P/F ratio, PaCO2
Guia et al. 2021 (Italy, Portugal, USA)^15^	ICU (COVID)	NIV (CPAP)	Need of IMV as rescue therapy and Death	5		27.3 %	23 %	HACOR score, Age, P/F ratio
Valencia et al. 2021 (Colombia)^13^	Emergency Department	HFNC (COVID)	Need of IMV as rescue therapy and Death	7.14 ±3.6	5.61 ±4.1	62.0 %	29.3 %	HACOR score, GCS, ROX index, Heart Rate, Respiratory Rate, PaCO2, SaO2, pH, PaO2, P/F ratio, Chronic Kidney Disease, Co-Infections
Jog S et al. 2021 (India)^20^	ICU (COVID)	NIV/HFNC	Need of IMV as rescue therapy and Death			64.1 %	56.9 %	Age, Medical Comorbidities, Admission SpO2, Non-Respiratory organ dysfunction
Prakash J et al. 2021 (Meta-analysis)^16^	ICU (COVID)	HFNC			5 (24 hr)			ROX score
Prakash J et al. 2021 (Meta-analysis)^16^	ICU (COVID)	HFNC			5 (24 hr)			ROX score
Khan MS et al., 2022 (India)^21^	ICU (COVID)	HFNC	Need of NIV or IMV as rescue therapy		3.4 ± 0.4 (1 hr) 4.4 ± 0.9 (6 hr)	28.9 %	27.1 % (28-day)	Age, APACHE ll, SOFA, Lag time to HFNC, Duration of HFNC, ICU LOS, PF ratio, ROX index, D-dimer, IL-6, RBS, Admission SpO2
Santus et al. 2022 (Italy)^14^	HDU (COVID)	CPAP (Helmet)	Need of IMV as rescue therapy, Transfer to ICU and Death	5 (1 hr)	6.2 (4.7–7.7) (1hr) mROX 5.8 (3.8–8.9) (1 hr)	38.4 %	12.4 %	Age, Ischaemic Heart Disease, COPD, CPAP duration, Hospital LOS, WBC count, D-dimer, CRP, PEEP, FiO2, PF ratio, Respiratory rate, A-a O2 gradient, GCS, ROX index, mROX index, HACOR score PaCO2,
Chacko et al. 2022 (India)^18^	ICU (COVID)	NIV	Need of IMV as rescue therapy or Death			36.4 %	30.1 %	Age, Disease Severity, Admission PF ratio, Respiratory Rate, High CK-MB, Need for organ support, Duration of continuous NIV, ICU LOS and hospital LOS

Age, SOFA and ROX D3 were significant factors on multivariate logistic regression model which predicted the failure, this seems logical as SOFA score basically covers each organ system. What additional factor ROX provides us is the RR, SOFA doesn’t provide. Age is important determinant as it decides two most important things host response to infection and above all body's cardiovascular response to hypoxia.

This is the first study to compare the ability to predict NIV failure using HACOR and ROX scores. A serial assessment of scores was done up to 6 days where none of the studies have assessed them for more than a day. Non-inferiority of ROX index, warrants future prospective trials on comparing these scores towards monitoring these patients in non-invasive ways. There is a need to redefine HACOR score for pts with hypoxemic resp failure as more focus should be paid on other parameters rather than traditionally described in HACOR scores such as SOFA, pneumonia, immunosuppression, ARDS and septic shock [11].

The limitation of this study may be thought off in terms of classical limitations associated with tendency of abstraction in a retrospective study, incomplete information of participants and changeability of medical professionals during patient dealing in different phases. However, all the attempts were made to minimize these possible sources of errors by defining research hypothesis a-priori, investigating through multitude of modalities like exploratory visualizations, univariable and multivariable analysis, inclusion of complete cases only in final analysis, running multiple step-up models and using multiple methods to determine convergence.

## Conclusion

The ROX score seems to be non-inferior to HACOR score in predicting NIV failure in patients with COVID-19 pneumonia, but this result should be interpreted with caution in other causes of acute respiratory failure. A cut of 4.47 at day-3 for ROX score consists of good discriminatory capacity to predicts NIV failure. From a multivariable perspective, older ages and SOFA score at admission were independent covariables with ROX score to predict NIV failure.
